# Socioeconomic inequality in the prevalence of noncommunicable diseases in low- and middle-income countries: Results from the World Health Survey

**DOI:** 10.1186/1471-2458-12-474

**Published:** 2012-06-22

**Authors:** Ahmad Reza Hosseinpoor, Nicole Bergen, Shanthi Mendis, Sam Harper, Emese Verdes, Anton Kunst, Somnath Chatterji

**Affiliations:** 1 Department of Health Statistics and Information Systems, World Health Organization, Geneva, Switzerland; 2 Department of Chronic Diseases and Health Promotion, World Health Organization, Geneva, Switzerland; 3 Department of Epidemiology, Biostatistics & Occupational Health, McGill University, Montreal, Canada; 4 Department of Public Health, AMC, University of Amsterdam, Amsterdam, Netherlands

## Abstract

**Background:**

Noncommunicable diseases are an increasing health concern worldwide, but particularly in low- and middle-income countries. This study quantified and compared education- and wealth-based inequalities in the prevalence of five noncommunicable diseases (angina, arthritis, asthma, depression and diabetes) and comorbidity in low- and middle-income country groups.

**Methods:**

Using 2002–04 World Health Survey data from 41 low- and middle-income countries, the prevalence estimates of angina, arthritis, asthma, depression, diabetes and comorbidity in adults aged 18 years or above are presented for wealth quintiles and five education levels, by sex and country income group. Symptom-based classification was used to determine angina, arthritis, asthma and depression rates, and diabetes diagnoses were self-reported. Socioeconomic inequalities according to wealth and education were measured absolutely, using the slope index of inequality, and relatively, using the relative index of inequality.

**Results:**

Wealth and education inequalities were more pronounced in the low-income country group than the middle-income country group. Both wealth and education were inversely associated with angina, arthritis, asthma, depression and comorbidity prevalence, with strongest inequalities reported for angina, asthma and comorbidity. Diabetes prevalence was positively associated with wealth and, to a lesser extent, education. Adjustments for confounding variables tended to decrease the magnitude of the inequality.

**Conclusions:**

Noncommunicable diseases are not necessarily diseases of the wealthy, and showed unequal distribution across socioeconomic groups in low- and middle-income country groups. Disaggregated research is warranted to assess the impact of individual noncommunicable diseases according to socioeconomic indicators.

## Background

The attenuation of the noncommunicable disease (NCD) burden has been cited as one of the greatest development challenges of the 21^st^ century [1,2]. In 2008, 80% of the world’s 36 million NCD-related deaths occurred in low- and middle-income countries (LMICs), which are home to most of the world’s population [3]. Globally, cardiovascular disease and diabetes are responsible for 170 million disability adjusted life years (11.3% of global burden), while cancers account for 78 million disability adjusted life years (5.1% of global burden), and respiratory diseases, 60 million disability adjusted life years (3.9% of global burden) [3]. Non-fatal NCDs, including depression [4] and arthritis [5] also contribute significantly to the global NCD burden. Without immediate and effective action, global NCD-related mortality is expected to reach 44 million in 2020, with a growing impact in low-resource settings [3].

NCDs are unequally distributed within populations, often disproportionally affecting the socioeconomically disadvantaged [3,6-9]. For example, a study of 52 countries at all stages of development found a trend for increased angina in poorer populations, although associations with individual- or societal-level socioeconomic markers were not analyzed [10]. A literature review reported elevated angina-related mortality and morbidity in less-affluent neighbourhoods, based on literature from several world regions [11]. Although few multinational studies of LMICs have examined the role of socioeconomic inequality in arthritis prevalence, preliminary research from the United States supported an association of low individual socioeconomic status with greater likelihood of arthritis in less-developed settings, and a potential role for community social determinants [12]. A lower level of formal education has been linked to higher arthritis prevalence [13-16], and worse arthritis outcomes [17] in high-income country settings. Studies that have analyzed associations between rates of asthma symptoms and country-level economic status have reported mixed findings [18-21], although greater asthma severity has been documented in populations of low socioeconomic status [20-22]. International depression studies universally report greater risk among women and populations of low economic standing or low levels of education [23,24], while diabetes was associated with low socioeconomic status in two high-income countries [25,26] and lower levels of education in middle- and high-income countries [8,27,28]. Previous studies from Southeast Asia reported associations between low education level and increased probability of having a chronic disease [29] and elevated number of chronic conditions [30].

In 2011, the international community convened at the United Nations High Level Summit to discuss approaches to address the rising trend of NCDs and their risk factors. In the absence of a concerted commitment amongst stakeholders, the international response thus far to alleviate this crisis has been deemed inadequate [31,32]. The amount of research dedicated to LMICs does not match the distribution of disease burden [33,34], and previous studies provide only a highly fragmented overview of the situation. Furthermore, prevention and treatment initiatives in low-resource settings are hindered by a lack of attention to social and economic situations [35].

The 2002–04 World Health Survey (WHS) gathered data about the symptoms of four major NCDs: angina, arthritis, asthma and depression as well as the self-reported diagnosis of diabetes. The objective of this study was to quantify and compare the socioeconomic inequalities in prevalence estimates of these five NCDs and comorbidity in LMICs.

## Methods

### Study population

Data were obtained from the 2002–04 WHS, conducted by the World Health Organization [36]. The WHS is a source of comparable population health data of adults aged 18 years and older in 70 countries from all regions of the world [37]. Survey samples were probabilistically selected, with all individuals having a known, non-zero chance of selection. WHS country surveys were nationally representative except in China, Comoros, Congo, Côte d'Ivoire, India, and the Russian Federation, where the WHS was carried out in geographically limited regions. To adjust for non-response and population distribution (as represented by the United Nations Statistical Division [38]), post-stratification corrections were made to sampling weights [4]. Informed consent was obtained in all surveys, using a procedure approved by institutional review boards [39]. The full list of local review boards from each study country is available in Additional file 1.

### Data

This study focused on LMICs, classified according to the World Bank’s development categories in 2003 [40], consistent with the timing of the majority of the WHS surveys. The 2002–04 WHS included 50 LMICs. Initially we assessed 48 LMICs that had available NCD prevalence data, and relevant socioeconomic and demographic data. (Guatemala did not have data on survey sampling weight, and Turkey had insufficient data to create the household wealth index, one of the principal variables of the study.) Seven countries were excluded from analysis because item non-response for any set of the questions pertaining to NCDs was over 20%. The final sample comprised 170,298 respondents (77,517 men and 92,781 women) from 41 LMICs. Household level response rates were over 70% in all study countries except Czech Republic. Individual level response rates were above 82%. See Additional file 2 for study country sample sizes, by sex, and Additional file 3, for NCD item non-response rates, by sex.

### Variables

We analyzed data for angina, arthritis, asthma, depression, and diabetes. These represent five of the six chronic conditions included in section 6000 of the WHS individual questionnaire, and prominent NCDs worldwide. (Psychosis/schizophrenia was not included as a study variable because its prevalence was too low to allow for meaningful disaggregated analysis by SES indicators, controlling for potential confounders.) Symptom-based classification was used to determine angina, arthritis, asthma and depression rates, and diabetes diagnoses were self-reported. The diagnostic criteria to estimate the prevalence of each disease has been previously described [4]. Briefly, angina was diagnosed based on an algorithm derived from the Rose questionnaire [41]. Arthritis and asthma diagnoses were based on responses to validated symptom-related questions [42-44]. Depression diagnosis was based on International Classification of Disease tenth revision: Diagnosis Criteria for Research for Depressive Episodes [45], and was derived from an algorithm that took into account depression symptoms [4,46]. For each diagnosis all potential answer combinations were considered, and the best result based on Receiver Operator Characteristic analysis within each country was applied as diagnosis criteria [47]. Diagnoses of these diseases were based on questions about the respondent’s condition in the 12 months preceding the interview date. For diabetes, diagnosis was based on a self-reported previous diagnosis. Comorbidity was defined as reporting of two or more NCDs by one individual. Because diabetes diagnosis was based solely on self-reported diagnosis (whereas symptom questions and the algorithm approach were used to diagnose the other NCDs), an additional comorbidity measure excluding diabetes was constructed.

Socioeconomic status was derived from household wealth status and individual highest-attained level of education. To measure household wealth, a dichotomous hierarchical ordered probit model was used to develop an index of the long-running economic status of households based on owning selected assets and/or using certain services [48-50]. The index was then divided into five quintiles within each country, with quintile one representing the poorest wealth quintile and quintile five, the richest. Education was ranked according to five categories: no formal schooling, less than primary school, primary school completed, secondary/high school completed, and college completed or above.

Confounders included sex, age (expressed categorically as 18–29, 30–39, 40–49, 50–59, 60–69 and 70 or more years), marital status (married/cohabiting, divorced/separated/widowed, or never married), area of residence (rural or urban), and country of residence.

### Methods of analysis

Prevalence rates for each NCD and comorbidity were calculated for men and women in LIC and MIC groups, according to wealth quintiles and education level. Both age-standardized [51] and crude prevalence estimates were calculated.

Socioeconomic inequality in each NCD and comorbidity prevalence was measured using the slope index of inequality (SII) and the relative index of inequality (RII), measures that take into account the distribution of the population across wealth quintiles or education levels [52]. Poisson regression model with a robust variance was used to assess the association between each NCD prevalence and socioeconomic status and to generate prevalence difference and prevalence ratio estimates, and 95% confidence intervals (95%CI) [53]. To calculate SII and RII, individuals were cumulatively ranked (ranging from zero to one) according to descending socioeconomic status (i.e. highest wealth or education level to lowest). The exposure variable can thus be interpreted as a continuous measure, with a value of zero equivalent to the top of the socioeconomic distribution and a value of one equivalent to the bottom. Therefore, SII is the prevalence rate difference--and RII, the prevalence rate ratio-- between those at top rank (representing the lowest level of wealth or education) and those at rank zero (representing the highest level of wealth or education). A SII value greater than zero and a RII value greater than one indicated an inverse gradient, where NCD prevalence was greater among populations of lower socioeconomic status. We referred to this situation as *"regular"* inequality, and conversely, *"reverse"* inequality if prevalence was higher among those with *higher* socioeconomic position [54]. Data were adjusted for country of residence and age (Model 1), as well as other confounding factors: marital status, urban/rural area and education or wealth (Model 2).

Stata® 11 was used for all analyses. We imputed missing values five times using Multiple Imputation by Integrated Chained Equations Technique [55]. All analyses were weighted, accounting for individual survey sample designs. The non-independence of observations within the surveys clusters were also incorporated in the analysis.

## Results

Table1 shows overall age-standardized prevalence of NCDs and comorbidity in men and women living in study LMICs. Overall, angina showed the highest prevalence rate. Overall age-standardized prevalence tended to be elevated in women. Except for depression, age-standardized prevalence rates were higher in MICs. Crude prevalence rates of NCDs and comorbidity are shown in Additional file 4.

**Table 1:** Age-standardized prevalence (%) of noncommunicable diseases among adults of 41 low- and middle-income countries, World Health Survey 2002-04

	**Men**						**Women**					
	**Middle-income group**			**Low-income group**			**Middle-income group**			**Low-income group**		
	**Estimate**	**95%CI**		**Estimate**	**95%CI**		**Estimate**		**95%CI**		**Estimate**	**95%CI**	
Angina	9.8	9.2	10.3	9.1	8.6	9.5	15.1	14.4	15.7	14.1	13.6	14.6
Arthritis	6.6	6.2	7.1	4.4	4.1	4.7	10.0	9.5	10.4	6.1	5.7	6.4
Asthma	6.4	6.0	6.8	5.5	5.1	5.9	6.8	6.4	7.2	6.4	6.0	6.8
Depression	3.9	3.6	4.2	4.9	4.5	5.2	7.0	6.5	7.4	7.8	7.4	8.2
Diabetes	3.7	3.3	4.0	1.4	1.2	1.6	5.0	4.7	5.3	1.5	1.3	1.7
Co-morbidity	6.3	5.9	6.7	4.6	4.3	4.9	10.1	9.6	10.6	7.1	6.7	7.4

### Wealth-related inequality

**Figure 1:**
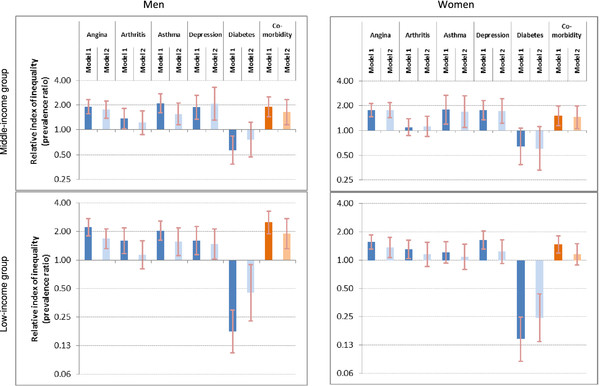
**Wealth-related relative inequality in non-communicable diseases among adults of 41 low- and middle-income countries.** The relative index of inequality shows wealth-related inequality in prevalence of angina, arthritis, asthma, depression, diabetes and comorbidity, among men and women aged 18 or higher, living in 41 low- and middle-income countries that participated in the 2002–04 World Health Survey. Individuals were cumulatively ranked by descending wealth, and prevalence ratios (RIIs) compared disease prevalence in the poorest to disease prevalence in the richest while taking into consideration all other individuals in the regression. Brackets indicate 95% confidence intervals. Model 1 data are adjusted for country of residence and age; model 2 data are adjusted for country of residence, age, marital status, urban/rural area and education.

Table2 summarizes age-standardized prevalence rates of NCDs and comorbidity by household wealth quintile and absolute inequalities (SII), among men and women living in study LMICs. Relative inequalities are illustrated in Figure1, and RII values are provided in Additional file 5. Additional file 6 summarizes crude prevalence of NCDs by wealth among each sex-income group.

**Table 2:** Noncommunicable disease prevalence (%) by wealth quintile, and wealth-related inequality among adults of 41 low- and middle-income countries, World Health Survey 2002-04

				**Angina**			**Arthritis**			**Asthma**			**Depression**			**Diabetes**			**Co-morbidity**		
				**Estimate**	**95% CI**		**Estimate**	**95% CI**		**Estimate**	**95% CI**		**Estimate**	**95% CI**		**Estimate**	**95% CI**		**Estimate**	**95% CI**	
Men	Middle-income group	Wealth quintile 1		13.0	11.9	14.1	6.9	6.1	7.7	9.5	8.4	10.6	5.5	4.7	6.2	2.7	2.2	3.2	8.3	7.4	9.1
		Wealth quintile 2		10.5	9.2	11.8	6.8	6.0	7.6	7.7	6.7	8.6	4.0	3.2	4.8	3.0	2.4	3.6	6.6	5.8	7.4
		Wealth quintile 3		9.8	8.8	10.8	6.5	5.8	7.2	6.6	5.8	7.4	3.9	3.3	4.6	4.1	3.5	4.7	6.3	5.5	7.0
		Wealth quintile 4		8.9	8.0	9.8	7.0	6.2	7.8	5.7	5.0	6.4	4.4	3.7	5.2	3.7	3.2	4.2	6.1	5.3	6.8
		Wealth quintile 5		6.9	6.0	7.8	5.1	4.3	5.8	4.1	3.5	4.7	2.3	1.8	2.8	4.3	3.8	4.9	4.1	3.4	4.8
		Slope index of inequality	Model 1*	7.3	5.0	9.6	2.4	0.3	4.5	5.4	3.4	7.5	2.5	1.1	3.8	-1.7	-2.8	-0.5	4.6	2.5	6.6
			Model 2**	6.4	3.7	9.1	1.5	-1.0	4.0	3.2	1.0	5.4	2.9	1.0	4.8	-0.8	-2.2	0.6	3.5	1.0	6.0
	Low-income group	Wealth quintile 1		10.8	9.9	11.6	4.5	3.9	5.1	6.7	5.9	7.5	5.7	5.0	6.4	0.7	0.4	1.0	5.7	5.1	6.4
		Wealth quintile 2		10.9	10.0	11.8	5.0	4.4	5.6	5.7	5.0	6.4	4.8	4.2	5.5	1.0	0.7	1.3	5.1	4.4	5.7
		Wealth quintile 3		9.3	8.4	10.2	4.2	3.7	4.7	5.9	5.2	6.7	4.8	4.1	5.4	1.2	0.9	1.4	4.9	4.2	5.5
		Wealth quintile 4		8.2	7.4	9.0	4.7	4.0	5.4	5.4	4.7	6.0	4.7	4.0	5.5	1.4	1.1	1.7	4.4	3.7	5.1
		Wealth quintile 5		6.6	5.7	7.4	3.7	3.2	4.3	4.4	3.8	5.0	4.1	3.5	4.7	2.5	2.1	2.9	3.3	2.7	3.9
		Slope index of inequality	Model 1*	7.8	5.9	9.8	2.2	0.8	3.7	4.1	2.7	5.4	2.5	0.8	4.2	-3.4	-4.5	-2.2	5.0	3.5	6.5
			Model 2**	5.0	2.8	7.3	0.6	-1.0	2.3	2.5	0.7	4.4	2.0	0.2	3.9	-1.4	-2.7	-0.2	3.5	1.6	5.4
Women	Middle-income group	Wealth quintile 1		18.6	17.4	19.9	9.6	8.7	10.4	9.2	8.4	10.1	9.1	8.2	10.0	4.2	3.6	4.9	12.2	11.3	13.1
		Wealth quintile 2		16.9	15.7	18.1	10.5	9.6	11.4	7.8	6.9	8.6	7.3	6.6	8.1	4.9	4.3	5.5	11.4	10.4	12.4
		Wealth quintile 3		15.6	14.4	16.8	10.1	9.3	11.0	7.1	6.3	7.8	7.0	6.1	7.8	4.6	4.1	5.1	10.4	9.5	11.3
		Wealth quintile 4		13.4	12.3	14.5	10.3	9.4	11.1	5.4	4.8	5.9	7.5	6.7	8.3	5.2	4.5	5.8	9.5	8.7	10.4
		Wealth quintile 5		11.9	10.8	12.9	9.8	8.9	10.7	5.6	5.0	6.2	5.1	4.4	5.8	5.6	4.9	6.3	8.3	7.5	9.2
		Slope index of inequality	Model 1*	10.6	7.2	13.9	1.0	-1.5	3.5	4.9	1.7	8.2	3.7	1.9	5.5	-2.2	-4.9	0.5	4.8	1.7	7.8
			Model 2**	10.4	6.7	14.2	1.2	-1.8	4.3	4.4	0.9	8.0	3.6	1.4	5.8	-2.5	-5.7	0.7	4.3	0.8	7.9
	Low-income group	Wealth quintile 1		15.4	14.4	16.3	6.3	5.6	7.0	6.4	5.7	7.1	8.6	7.7	9.4	0.6	0.4	0.9	7.3	6.6	8.0
		Wealth quintile 2		15.7	14.7	16.8	6.6	5.9	7.2	7.4	6.6	8.2	8.5	7.7	9.3	1.0	0.7	1.2	7.9	7.2	8.7
		Wealth quintile 3		14.0	12.9	15.2	6.8	6.0	7.5	6.5	5.7	7.3	8.2	7.4	9.1	1.1	0.8	1.4	7.4	6.6	8.2
		Wealth quintile 4		13.2	12.1	14.3	5.8	5.1	6.4	5.8	5.1	6.5	7.6	6.8	8.4	1.7	1.3	2.0	6.4	5.7	7.1
		Wealth quintile 5		11.9	10.6	13.2	5.5	4.7	6.3	5.9	5.2	6.6	5.8	5.0	6.6	3.2	2.7	3.7	6.3	5.5	7.2
		Slope index of inequality	Model 1*	7.0	4.3	9.7	2.0	0.3	3.6	1.1	-0.4	2.6	4.2	2.3	6.2	-4.0	-5.3	-2.8	3.1	1.4	4.8
			Model 2**	4.9	1.1	8.7	1.1	-1.1	3.2	0.5	-1.3	2.2	1.8	-0.7	4.3	-2.7	-3.9	-1.6	1.2	-0.9	3.3

* Model 1 is adjusted for country of residence and age.

** Model 2 is adjusted for country of residence, age, marital status, urban/rural area and education.

We generally found regular inequality in prevalence values of angina, arthritis, asthma and depression in both absolute and relative terms, after controlling for respondents' age and country of residence (Model 1). Of all NCDs, angina consistently demonstrated the highest absolute inequality. Notably, the absolute difference of angina prevalence between poorest and richest women of the MIC group was over 10% points (prevalence difference: 10.6%, 95%CI 7.2%-13.9%). Arthritis demonstrated the weakest inequality, and was non-significant after controlling for other study confounders (Model 2). Diabetes prevalence was positively associated with increasing wealth quintile. This reverse inequality was more pronounced in the LIC group, and lost significance in MICs when data were adjusted for confounders (Model 2). For example, diabetes prevalence in the poorest women living in the study LIC group was one fourth of the richest in Model 2 (prevalence ratio: 0.24, 95%CI 0.14-0.44).

Comorbidity prevalence showed an inverse association with wealth quintile in all four sex-income groups; i.e. regular inequality. In both country income groups comorbidity was around 1.5 times more prevalent in the poorest women than in the richest in Model 1 (prevalence ratio: women of study LICs: 1.46, 95%CI 1.19-1.80; women of study MICs: 1.51, 95%CI 1.14-1.98). Comorbidity was 2.5 times more prevalent in the poorest men of study LICs than in the richest (prevalence ratio: 2.50, 95%CI 1.89-3.30). Wealth-related inequalities in comorbidity remained statistically significant in Model 2 in all sex-income groups except for women living in the LIC group. Excluding diabetes from the comorbidity measure strengthened regular inequality, which was robust to adjustment for confounding.

### Education-related inequality

**Figure 2:**
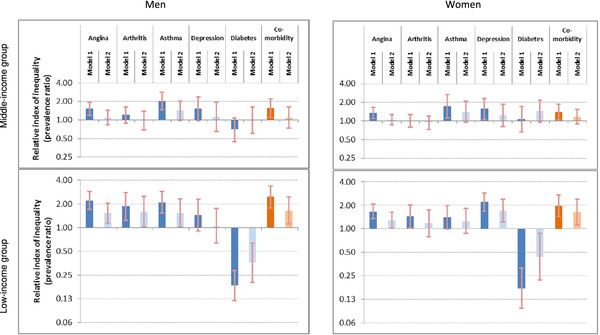
**Education-related relative inequality in non-communicable diseases among adults of 41 low- and middle-income countries.** The relative index of inequality shows education-related inequality in prevalence of angina, arthritis, asthma, depression, diabetes and comorbidity, among men and women aged 18 or higher, living in 41 low- and middle-income countries that participated in the 2002–04 World Health Survey. Individuals were cumulatively ranked by descending education level, and prevalence ratios (RIIs) compared disease prevalence in the least educated group to disease prevalence in the most educated group while taking into consideration all other groups in the regression. Brackets indicate 95% confidence intervals. Model 1 data are adjusted for country of residence and age; model 2 data are adjusted for country of residence, age, marital status, urban/rural area and wealth.

Table3 summarizes age-standardized prevalence rates of NCDs and comorbidity by education and absolute inequalities (SII), among men and women living in study LMICs. Relative inequalities are illustrated in Figure2, and RII values are provided in Additional file 7. Additional file 8 summarizes crude prevalence of NCDs by education among each sex-income group.

**Table 3:** Noncommunicable disease prevalence (%) by education level, and education-related inequality among adults of 41 low- and middle-income countries, World Health Survey 2002-04

				**Angina**			**Arthritis**			**Asthma**			**Depression**			**Diabetes**			**Co-morbidity**		
				**Estimate**	**95% CI**		**Estimate**	**95% CI**		**Estimate**	**95% CI**		**Estimate**	**95% CI**		**Estimate**	**95% CI**		**Estimate**	**95% CI**	
Men	Middle-income group	No formal schooling		12.0	10.4	13.7	7.9	6.4	9.3	9.2	7.7	10.8	7.1	5.8	8.3	2.7	1.9	3.4	7.7	6.4	9.0
		Less than primary school		10.5	9.3	11.8	7.9	6.7	9.0	6.8	5.9	7.8	5.2	4.2	6.1	3.7	3.0	4.4	7.8	6.7	9.0
		Primary school completed		10.9	9.7	12.1	6.0	5.2	6.8	7.4	6.5	8.2	5.2	4.4	6.0	4.3	3.8	4.9	7.4	6.5	8.2
		Secondary/high school completed		8.5	7.8	9.2	6.1	5.5	6.7	5.7	5.1	6.3	3.2	2.7	3.7	4.1	3.6	4.7	5.2	4.7	5.8
		College completed or above		6.6	5.7	7.4	5.5	4.6	6.4	4.2	3.0	5.3	2.6	1.9	3.3	4.0	3.3	4.7	4.3	3.7	4.9
		Slope index of inequality	Model 1*	4.8	2.0	7.5	1.4	−1.0	3.8	5.3	2.7	7.8	1.6	−0.1	3.3	−1.1	−2.5	0.3	3.1	0.7	5.6
			Model 2**	1.0	−2.1	4.0	−0.2	−2.9	2.5	2.5	−0.1	5.1	0.5	−1.6	2.6	0.0	−1.5	1.4	0.6	−2.1	3.4
	Low-income group	No formal schooling		10.2	9.4	11.1	5.2	4.6	5.8	6.4	5.7	7.1	5.5	4.8	6.1	0.8	0.6	1.0	5.5	4.9	6.1
		Less than primary school		10.1	9.1	11.1	4.2	3.6	4.7	5.7	4.8	6.5	4.9	4.2	5.7	1.4	1.0	1.8	5.1	4.4	5.8
		Primary school completed		8.2	7.3	9.1	3.4	2.8	3.9	6.0	5.0	6.9	4.4	3.6	5.1	1.6	1.2	2.1	3.9	3.2	4.5
		Secondary/high school completed		6.4	5.6	7.3	3.5	2.8	4.2	4.6	3.8	5.3	3.8	3.1	4.6	2.6	2.1	3.2	3.0	2.5	3.6
		College completed or above		4.4	3.2	5.7	3.0	2.1	4.0	3.7	2.8	4.7	4.1	3.0	5.1	2.8	2.0	3.5	2.5	1.7	3.3
		Slope index of inequality	Model 1*	7.6	5.2	10.0	2.9	1.1	4.7	4.1	2.3	5.9	1.9	−0.4	4.2	−3.7	−4.9	−2.4	4.8	3.1	6.5
			Model 2**	4.1	1.4	6.9	2.2	0.2	4.2	2.4	0.2	4.6	0.3	−2.4	3.0	−2.0	−3.2	−0.8	2.7	0.7	4.7
Women	Middle-income group	No formal schooling		17.9	16.4	19.4	10.7	9.6	11.8	10.2	9.0	11.4	12.0	10.5	13.5	7.7	6.7	8.7	15.2	13.9	16.5
		Less than primary school		17.0	15.2	18.9	8.8	7.9	9.7	9.7	8.8	10.6	7.0	5.9	8.0	5.7	5.1	6.4	11.6	10.7	12.6
		Primary school completed		16.2	14.8	17.5	11.8	10.7	12.9	7.7	6.7	8.6	7.6	6.2	8.9	6.3	5.6	7.0	11.9	10.7	13.1
		Secondary/high school completed		13.3	12.5	14.2	8.8	8.1	9.5	6.2	5.7	6.8	5.7	5.0	6.4	5.1	4.5	5.7	8.8	8.1	9.5
		College completed or above		9.5	8.5	10.4	8.8	7.9	9.6	3.9	3.3	4.6	5.1	4.3	6.0	2.9	2.4	3.5	5.6	4.9	6.3
		Slope index of inequality	Model 1*	5.6	1.9	9.3	0.0	−2.6	2.6	4.7	1.1	8.4	3.0	0.5	5.5	0.3	−2.0	2.6	3.8	0.5	7.2
			Model 2**	0.9	−2.7	4.6	−0.7	−3.5	2.0	2.9	−0.4	6.2	1.4	−1.3	4.1	1.7	−0.1	3.6	1.9	−1.2	5.1
	Low-income group	No formal schooling		14.7	13.9	15.5	6.1	5.7	6.6	7.1	6.5	7.7	8.4	7.8	9.0	1.1	0.9	1.3	7.7	7.1	8.2
		Less than primary school		15.1	13.9	16.3	6.4	5.4	7.5	5.7	5.0	6.4	7.4	6.5	8.3	2.6	2.0	3.3	7.1	6.2	8.0
		Primary school completed		11.6	10.2	13.0	5.4	4.5	6.3	6.2	5.2	7.2	5.9	5.0	6.8	2.2	1.7	2.7	5.4	4.5	6.3
		Secondary/high school completed		10.6	9.4	11.9	5.9	5.0	6.8	5.5	4.7	6.4	6.2	5.1	7.3	3.2	2.5	4.0	6.6	5.6	7.7
		College completed or above		9.0	7.5	10.6	3.9	2.9	4.9	3.1	1.9	4.3	3.4	2.2	4.5	3.5	2.6	4.4	3.9	2.9	4.9
		Slope index of inequality	Model 1*	7.8	4.6	11.0	2.6	0.2	5.0	1.9	−0.1	3.8	6.6	4.3	8.9	−4.6	−6.7	−2.5	5.3	2.9	7.8
			Model 2**	3.8	0.2	7.5	1.2	−1.7	4.1	1.3	−0.8	3.4	4.5	1.7	7.3	−1.8	−3.4	−0.1	3.9	1.0	6.8

* Model 1 is adjusted for country of residence and age.

** Model 2 is adjusted for country of residence, age, marital status, urban/rural area and wealth.

Controlling for age and country of residence, regular inequalities were reported for the prevalence of angina and asthma in most study populations. Education-related inequality was weak or non-significant for arthritis prevalence (except among men of LICs), and also for depression prevalence (except among women of LICs). Depression was twice as prevalent among LIC women with no formal education as women with college/university education (prevalence ratio: 2.19, 95%CI 1.68-2.87). The strongest education-related regular inequality was found among LIC populations for angina. The absolute difference of angina prevalence between adults with no formal education and those with college/university education was near 8% points (prevalence difference: men: 7.6%, 95%CI 5.2%-10.0%; women: 7.8%, 95%CI 4.6%-11.0%). In MICs, all education-related inverse associations were non-significant after adjusting for other confounding factors (Model 2). Diabetes prevalence was associated with increasing education level (reverse inequality), demonstrating a significant relationship in LICs, after adjusting for all confounding factors (prevalence ratio: men: 0.36, 95%CI 0.20-0.64; women: 0.44, 95% CI 0.22-0.88).

Comorbidity showed regular inequality, with an inverse association between comorbidity prevalence and education level in all sex-income groups. The absolute difference in comorbidity prevalence among adults living in the LIC group was about 5% points across educational levels in Model 1. Education-related inequality was robust to adjustment for confounding in the LIC group, but not the MIC group.

## Discussion

Within LICs and MICs, wealth- and education-related inequalities of variable magnitudes and direction were quantified for the five NCDs. In most populations, regular inequalities in terms of wealth and education were reported for angina, arthritis, asthma and depression, with the strongest associations for angina, asthma and comorbidity. For all NCDs, additional adjustments for confounding factors in Model 2 (marital status, urban/rural area and wealth/education) tended to decrease the magnitude of inequality, and may thus help to explain disparities in NCD prevalence.

These findings are in accordance with previous reports from a variety of settings, which also reported inverse associations between socioeconomic position and prevalence of angina [10,11,56], arthritis [12,13,15,16], asthma [57,58], and depression [23,24,59-62]. Previously, European data (including eight higher-income countries) from the 1990s demonstrated education-related inequality in 14 of 17 studied NCDs, most notably stroke, diseases of the nervous system and diabetes. There was a tendency for stronger inequality in adults aged 25–59 than those aged 60–79 [9]. Goyal et al. (2010) highlighted differences based on country income group, reporting that education had more of a protective effect against cardiovascular events in high-income countries than LMICs, and also among men [27].

The connection between socioeconomic status and health is complex, and shaped by diverse circumstantial factors as well as political, social and economic forces [63]. For example, people living in poverty may experience material deprivation and high stress levels, which may lead to constrained choices and a higher likelihood of engaging in risky health behaviours, increasing the risk of disease; following disease onset, reduced access to care hinders opportunities to prevent complications [64]. It has been estimated that up to 80% of cases of cardiovascular disease or type 2 diabetes and 40% of cancer cases are preventable based on current knowledge, however, prevention initiatives may not adequately reach vulnerable populations where disease risk factors cluster [65].

Unlike the other four NCDs, we reported higher diabetes prevalence among the wealthier and more educated, especially in LICs. These findings conflict with trends reported by previous studies conducted in higher-income countries [25,26]. Epidemiological studies in lower-income countries are less-forthcoming, however diabetes was reported to be positively associated with affluence in the Dominican Republic [66], and metabolic syndrome was positively associated with affluence among adolescents in India [67]. Nations at different levels of development may realize different stages of disease epidemiological transitions [67]. Noting methodological differences in determining diabetes prevalence, it is also possible that our findings may be subjected to bias stemming from a methodology issue whereby cases with a lower wealth or education were more likely to be under-diagnosed and therefore prevalence rates were underestimated. Populations in less-developed nations may have limited access to medical professionals [6], which could result in under-diagnosis of diabetes, particularly among populations of lower socio-economic status; for example, better educated individuals may be more aware of diabetes as a health condition. Alternatively, this finding may reflect a complex relationship between wealth, overweight, obesity, other risk factors (such as physical inactivity), and diabetes [68]. Ideally, future surveys may integrate objective indicators of disease, such as HbA1C diagnostic testing for diabetes [69].

Comorbidity significantly lowers quality of life, affecting physical, social and psychological well-being [70]. Our findings showed that comorbidity was more prevalent among the poor and less educated, in all sex-income groups. We reported overall comorbidity rates of up to 10%, with even greater prevalence in some poorer wealth quintiles and least educated subpopulations. In a multinational study of high-income countries 30.2% of adults over 18 reported more than one chronic condition, although the study included seven diseases whereas ours included five [71]. Consistent with the present study, previous research has reported inverse associations between comorbidity and markers of socioeconomic status [30].

Like other studies, NCD prevalence tended to be higher in women than men [30], and the greatest burden was reported for a cardiovascular-related condition [3]. Overall, study NCDs tended to be more prevalent in the MIC group, with the exception of depression. That depression rates were higher in LICs than MICs was not expected. Previously, depression prevalence was reported to be lower in a less-developed setting, although cultural willingness to report depressive symptoms may bias outcomes [72]. According to epidemiological transition models, LICs may be expected to carry a lower-- albeit increasing-- burden of NCDs than higher-income country groups, as risk factors for infectious diseases are progressively replaced by risk factors for NCDs [73]. Projections for 2005–2030 forecasted a 10% increase in the deaths due to chronic disease in LMICs (from 61% to 71%) [74]. Monitoring trends in NCD prevalence in LMIC groups will help to characterize the nature of the modern epidemiological transition, and identify populations that are most at risk for NCDs.

### Strengths, limitations and implications

Data for five NCDs were collected systematically in a large sample of LMICs that participated in the WHS, allowing for comparisons of standardized data across pooled data sets. Consistent diagnostic criteria in WHS data facilitated broad-scale analyses and comparisons across several countries, minimizing limitations associated with variable measurement tools and disease classifications. However, the use of pooled data from geographically- and culturally-diverse settings inevitably masks problems of comparability between countries [54]. Nine studies were excluded from analysis due to insufficient data or high item non-response rates. There is no reason to believe that the excluded countries would have changed the main findings on socioeconomic inequality in LMICs. The non-response was not selective, including countries of both low and middle income groups. We included a country variable in our multivariate analysis in order to control for any potential confounding effect of the individual countries. We did not aim to explore the interaction effects of our study’s independent variables with each of the countries.

Wealth and education levels were determined nationally, and pooled across LICs and MICs. We acknowledge that patterns of wealth distribution vary between countries, however, quintile classification provided a widely accepted method to compare respondents based on relative wealth position within their country. Levels of education were standardized to be comparable across countries.

The use of symptom-based diagnoses for angina, arthritis, asthma and depression was a strength, as other methods that rely on medical charts or self-reported diagnoses may introduce biases related to health system access. Self-reported data could reflect systematic over- or under-reporting, which may vary by socioeconomic status [75]. A tendency for under-reporting of symptoms by people with low levels of education [76] raises the possibility that our data may underestimate prevalence in low education classes, and show weaker-than-actual inverse associations. As a result, our data may underestimate true NCD rates in socioeconomic disadvantaged populations.

It is possible that a selection bias may have occurred in the sampling process, especially in countries with lower response rate, although we are not aware of evidence to suggest that this had occurred. The main reasons for household non-response included inability to locate the selected household, or household refusal to participate even before a roster could be obtained.

## Conclusions

Action taken in the next 20 years will be critical in determining the outcome of the mounting NCD epidemic [77]. One of the major reasons for the relative failure of NCD advocacy is a lack of emphasis on social justice and inequality [78]. Delineating the impact of NCDs on poor and rich populations-- both between country-income groupings and within countries --is an important precursor to NCD prevention and management efforts.

The focus of this study was to assess within-country socioeconomic distribution of five NCDs and comorbidity in LMICs. We reported disparities between subpopulations of different wealth- and education-levels, which varied according to the type of NCD. With the exception of diabetes, four NCDs and their comorbidity showed some evidence for unequal distribution in populations, to the detriment of those with lower wealth or education levels. Angina, asthma and comorbidity prevalence demonstrated the strongest inverse associations with wealth and education; arthritis and depression also reported inverse wealth and education associations in most cases. In LICs, diabetes prevalence was significantly positively associated with wealth and education.

Overall, our mixed findings substantiate the need for disaggregated research to delineate the impact of individual NCDs on various socioeconomic groups. High quality epidemiological evidence is a cornerstone of effective policy development, deployment and monitoring. The present study has shown that NCDs are not necessarily diseases of the wealthy, demonstrating unequal distribution across socioeconomic groups. Further investigation of the risk factors and root causes of socioeconomic inequality are warranted to formulate sustainable and effective approaches to prevent and manage NCDs among the poor and low educated.

## Abbreviations

LICs: Low-income countries; LMICs: Low- and middle-income countries; MICs: Middle-income countries; NCDs: Noncommunicable diseases; RII: Relative index of inequality; SII: Slope index of inequality; WHS: World Health Survey.

## Competing interests

The authors declare that they have no competing interests.

## Authors’ contributions

AH designed the study. AH did the statistical analysis with inputs from EV. NB wrote the first draft with inputs from AH. AK, SH, SM, SC read the draft and provided critical comments. All authors read and approved the final draft.

## Pre-publication history

The pre-publication history for this paper can be accessed here:

http://www.biomedcentral.com/1471-2458/12/474/prepub

## Supplementary Material

Additional file 1 **Local review boards for study countries.** Lists each study country and its corresponding local review board. Click here for file

Additional file 2 **Study sample size by country and sex, World Health Survey 2002–04.** Displays the study sample size of men and women (aged 18 or higher) from 41 low- and middle-income countries that participated in the 2002–04 World Health Survey. Click here for file

Additional file 3 **Noncommunicable diseases non-response rates, by country and sex, World Health Survey 2002–04.** Displays the non-response rates to World Health Survey individual questionnaires for each studied noncommunicable disease, grouped by sex and low- or middle-income country status. Data represent 41 low- and middle-income countries that participated in the 2002–04 World Health Survey. Click here for file

Additional file 4 **Crude prevalence (%) of noncommunicable diseases among adults aged 18 or higher living in 41 low- and middle-income countries, World Health Survey 2002–04.** Displays the crude prevalence rates (percentage) of each studied noncommunicable disease and comorbidity among adults (aged 18 or higher), grouped by sex and low- or middle-income country status. Data represent 41 low- and middle-income countries that participated in the 2002–04 World Health Survey. Click here for file

Additional file 5 **Wealth-related relative inequality in noncommunicable disease prevalence among adults aged 18 or higher living in 41 low- and middle-income countries, World Health Survey 2002–04.** Displays the relative index of inequality and corresponding 95% confidence interval for each studied noncommunicable disease and comorbidity among adults (aged 18 or higher), according to wealth quintile. Data are grouped by sex and low- or middle-income country status, and represent 41 low- and middle-income countries that participated in the 2002–04 World Health Survey. Model 1 data are adjusted for country of residence and age; model 2 data are adjusted for country of residence, age, marital status, urban/rural area and education. Click here for file

Additional file 6 **Crude prevalence (%) of non-communicable diseases among adults aged 18 or higher living in 41 low- and middle-income countries, by wealth, World Health Survey 2002–04.** Displays the crude prevalence rates (percentage) of each studied noncommunicable disease and comorbidity among adults (aged 18 or higher), according to wealth quintile. Data are grouped by sex and low- or middle-income country status, and represent 41 low- and middle-income countries that participated in the 2002–04 World Health Survey. Click here for file

Additional file 7 **Education-related relative inequality in noncommunicable disease prevalence among adults aged 18 or higher living in 41 low- and middle-income countries, World Health Survey 2002–04.** Displays the relative index of inequality and corresponding 95% confidence interval for each studied noncommunicable disease and comorbidity among adults (aged 18 or higher), according to education level. Data are grouped by sex and low- or middle-income country status, and represent 41 low- and middle-income countries that participated in the 2002–04 World Health Survey. Model 1 data are adjusted for country of residence and age; model 2 data are adjusted for country of residence, age, marital status, urban/rural area and wealth. Click here for file

Additional file 8 **Crude prevalence (%) of noncommunicable diseases among adults aged 18 or higher living in 41 low- and middle-income countries, by education, World Health Survey 2002–04.** Displays the crude prevalence rates (percentage) of each studied noncommunicable disease and comorbidity among adults (aged 18 or higher), according to education level. Data are grouped by sex and low- or middle-income country status, and represent 41 low- and middle-income countries that participated in the 2002–04 World Health Survey. Click here for file
