# Supplementary material for: Socioeconomic inequality in the prevalence of noncommunicable diseases in low- and middle-income countries: Results from the World Health Survey
Source: BMC Public Health. 2012 Jun 22;12:474. doi: 10.1186/1471-2458-12-474 (PMC3490890; doi:10.1186/1471-2458-12-474)
Supplement: Additional file 1 — Local review boards for study countries. Lists each study country and its corresponding local review board. [file 1471-2458-12-474-S1.pdf]

Additional file 1: Local review boards for study countries

Study Country	Local Review Board
Bangladesh	Mitra and Associates
Bosnia and Herzegovina	The Federal Public Health Institute
Burkina Faso	Institut de Recherche en Sciences de la Santé
Chad	Faculté des Sciences de la Santé, Univ N'Djamena
China	Centre for Health Statistics Information
Comoros	Bureau Comorien de Conseil
Cote d'Ivoire	Ministère de la Santé
Croatia	The Croatian National Institute of Public Health
Czech Republic	Institute of Health Information and Statistics
Dominican Republic	Centro de Estudios Sociales y Demográficos (CESDEM)
Ecuador	Fundación Ecuatoriana para la Salud y el Desarroll (FESALUD)
Estonia	Saar Poll Ltd.
Ethiopia	Department of Community Health, Jimma University
Georgia	Georgian State Medical Academy (GSMA)
Ghana	Department of Community Health, Ghana Medical Sch
India	International Institute of Population Sciences
Kazakhstan	Kazakstan School of Public Health (KSPH)
Kenya	Central Bureau of Statistics
Lao People's Democratic Republic	National Institute of Public Health, Ministry of Health
Latvia	The Health Promotion Center
Malawi	Centre for Social Research (CSR)
Malaysia	Public Health Institute, Ministry of Health
Mali	Cellule de Planification et de Statistique, (CPS)
Mauritania	Office Nationale de la Statistique (ONS)
Mauritius	Mauritius Instituteof Health
Morocco	Ministère de la Santé
Myanmar	Department of Medical Research, Ministry of Health
Namibia	Ministry of Health
Pakistan	Ministry of Health
Paraguay	Fac.de Ciencias Veterinarias, Univ. Nacional/DGEEC
Philippines	College of Medicine, University of the Philippines
Russian Federation	Semashko Institute for Research on Social Hygiene,
Senegal	Direction Etudes, Recherche et Formation (DERF)
South Africa	Community Agency for Social Enquiry (CASE)
Sri Lanka	Ministry of Health
Tunisia	Institut National de la Santé Publique
Ukraine	Odessa State Medical University
Uruguay	Centro de Estudios de Economia y Salud (CEES)
Viet Nam	Ministry of Health
Zambia	School of Humanities & Social Sciences, University of Zambia
Zimbabwe	Community Health, University of Zimbabwe
