# Supplementary material for: Socioeconomic inequality in the prevalence of noncommunicable diseases in low- and middle-income countries: Results from the World Health Survey
Source: BMC Public Health. 2012 Jun 22;12:474. doi: 10.1186/1471-2458-12-474 (PMC3490890; doi:10.1186/1471-2458-12-474)
Supplement: Additional file 2 — Study sample size by country and sex, World Health Survey 2002–04. Displays the study sample size of men and women (aged 18 or higher) from 41 low- and middle-income countries that participated in the 2002–04 World Health Survey. [file 1471-2458-12-474-S2.pdf]

Additional file 2: Study sample size, by country and sex, World Health Survey 2002-04

	Sample size		
	Total	Men	Women
**Middle-income countries**	**83,063**	**36,558**	**46,505**
Bosnia and Herzegovina	1,028	434	594
China	3,993	1,954	2,039
Croatia	990	401	589
Czech Republic	935	419	516
Dominican Republic	4,534	2,104	2,430
Ecuador	4,608	2,043	2,565
Estonia	1,011	367	644
Georgia	2,749	1,162	1,587
Kazakhstan	4,496	1,544	2,952
Latvia	855	286	569
Malaysia	6,038	2,671	3,367
Mauritius	3,888	1,872	2,016
Morocco	4,472	2,074	2,398
Namibia	4,246	1,722	2,524
Paraguay	5,131	2,348	2,783
Philippines	10,075	4,659	5,416
Russian Federation	4,422	1,593	2,829
South Africa	2,351	1,115	1,236
Sri Lanka	6,698	3,117	3,581
Tunisia	5,068	2,343	2,725
Ukraine	2,498	883	1,615
Uruguay	2,977	1,447	1,530
**Low-income countries**	**87,235**	**40,959**	**46,276**
Bangladesh	5,550	2,584	2,966
Burkina Faso	4,821	2,271	2,550
Chad	4,635	2,191	2,444
Comoros	1,758	786	972
Cote d'Ivoire	3,178	1,815	1,363
Ethiopia	4,936	2,390	2,546
Ghana	3,931	1,772	2,159
India	9,723	4,705	5,018
Kenya	4,346	1,847	2,499
Lao People's Democratic Republic	4,888	2,295	2,593
Malawi	5,297	2,210	3,087
Mali	4,145	2,382	1,763
Mauritania	3,705	1,440	2,265
Myanmar	5,886	2,551	3,335

Pakistan	6,104	3,402	2,702
Senegal	2,970	1,548	1,422
Viet Nam	3,491	1,571	1,920
Zambia	3,810	1,722	2,088
Zimbabwe	4,061	1,477	2,584
