# Supplementary material for: Socioeconomic inequality in the prevalence of noncommunicable diseases in low- and middle-income countries: Results from the World Health Survey
Source: BMC Public Health. 2012 Jun 22;12:474. doi: 10.1186/1471-2458-12-474 (PMC3490890; doi:10.1186/1471-2458-12-474)
Supplement: Additional file 3 — Noncommunicable diseases non-response rates, by country and sex, World Health Survey 2002–04. Displays the non-response rates to World Health Survey individual questionnaires for each studied noncommunicable disease, grouped by sex and low- or middle-income country status. Data represent 41 low- and middle-income countries that participated in the 2002–04 World Health Survey. [file 1471-2458-12-474-S3.pdf]

Additional file 3: Noncommunicable diseases non-response rates, by country and sex, World Health Survey 2002-04

	Non-response rate (%)									
	Men					Women				
	Angina	Arthritis	Asthma	Depression	Diabetes	Angina	Arthritis	Asthma	Depression	Diabetes
Middle-income countries	7	3	2	8	2	8	3	2	9	2
Low-income countries	8	4	3	7	3	10	3	3	7	3
