# Supplementary material for: Socioeconomic inequality in the prevalence of noncommunicable diseases in low- and middle-income countries: Results from the World Health Survey
Source: BMC Public Health. 2012 Jun 22;12:474. doi: 10.1186/1471-2458-12-474 (PMC3490890; doi:10.1186/1471-2458-12-474)
Supplement: Additional file 4 — Crude prevalence (%) of noncommunicable diseases among adults aged 18 or higher living in 41 low- and middle-income countries, World Health Survey 2002–04. Displays the crude prevalence rates (percentage) of each studied noncommunicable disease and comorbidity among adults (aged 18 or higher), grouped by sex and low- or middle-income country status. Data represent 41 low- and middle-income countries that participated in the 2002–04 World Health Survey. [file 1471-2458-12-474-S4.pdf]

Additional file 4: Crude prevalence (%) of noncommunicable diseases among adults aged 18 or higher living in 41 low- and middle-income countries, World Health Survey 2002-04

	Men						Women					
	Middle-income group			Low-income group			Middle-income group			Low-income group		
	Estimate	95%CI		Estimate	95%CI		Estimate	95%CI		Estimate	95%CI	
Angina	7.0	6.6	7.5	9.9	9.2	10.6	18.8	17.7	19.9	15.5	14.7	16.4
Arthritis	7.7	7.0	8.3	4.8	4.3	5.2	11.0	10.2	11.7	7.3	6.8	7.9
Asthma	7.4	6.7	8.1	5.7	5.2	6.2	8.6	7.9	9.4	5.7	5.3	6.2
Depression	3.9	3.5	4.4	5.3	4.7	5.8	6.7	6.0	7.3	8.7	8.0	9.3
Diabetes	2.9	2.6	3.3	1.9	1.6	2.1	4.8	4.3	5.4	2.0	1.7	2.3
Co-morbidity	7.2	6.6	7.8	5.5	5.0	6.0	11.8	10.9	12.7	8.2	7.6	8.7
