# Supplementary material for: Socioeconomic inequality in the prevalence of noncommunicable diseases in low- and middle-income countries: Results from the World Health Survey
Source: BMC Public Health. 2012 Jun 22;12:474. doi: 10.1186/1471-2458-12-474 (PMC3490890; doi:10.1186/1471-2458-12-474)
Supplement: Additional file 5 — Wealth-related relative inequality in noncommunicable disease prevalence among adults aged 18 or higher living in 41 low- and middle-income countries, World Health Survey 2002–04. Displays the relative index of inequality and corresponding 95% confidence interval for each studied noncommunicable disease and comorbidity among adults (aged 18 or higher), according to wealth quintile. Data are grouped by sex and low- or middle-income country status, and represent 41 low- and middle-income countries that participated in the 2002–04 World Health Survey. Model 1 data are adjusted for country of residence and age; model 2 data are adjusted for country of residence, age, marital status, urban/rural area and education. [file 1471-2458-12-474-S5.pdf]

Additional file 5: Wealth-related relative inequality in noncommunicable disease prevalence among adults aged 18 or higher living in 41 low- and middle-income countries, World Health Survey 2002-04

		Men						Women					
		Middle-income group			Low-income group			Middle-income group			Low-income group		
		Estimate	95%CI		Estimate	95%CI		Estimate	95%CI		Estimate	95%CI	
Angina	Model 1	1.90	1.56	2.32	2.23	1.81	2.74	1.77	1.47	2.13	1.57	1.32	1.87
	Model 2	1.76	1.39	2.23	1.68	1.32	2.14	1.75	1.42	2.17	1.37	1.07	1.76
Arthritis	Model 1	1.37	1.03	1.82	1.60	1.17	2.20	1.09	0.87	1.38	1.31	1.05	1.64
	Model 1	1.22	0.88	1.70	1.14	0.81	1.61	1.12	0.84	1.48	1.15	0.86	1.55
Asthma	Model 2	2.10	1.61	2.73	2.05	1.63	2.58	1.79	1.19	2.68	1.21	0.94	1.57
	Model 1	1.56	1.15	2.10	1.57	1.12	2.19	1.69	1.08	2.62	1.08	0.80	1.47
Depression	Model 1	1.87	1.33	2.63	1.61	1.14	2.26	1.75	1.34	2.29	1.63	1.31	2.04
	Model 2	2.08	1.31	3.31	1.47	1.02	2.12	1.72	1.22	2.42	1.24	0.92	1.66
Diabetes	Model 1	0.57	0.38	0.84	0.18	0.11	0.30	0.64	0.38	1.06	0.14	0.08	0.25
	Model 2	0.76	0.47	1.23	0.45	0.23	0.90	0.60	0.33	1.10	0.24	0.14	0.44
Co-morbidity	Model 1	1.90	1.44	2.53	2.50	1.89	3.30	1.51	1.14	1.98	1.46	1.19	1.80
	Model 2	1.65	1.16	2.34	1.91	1.33	2.74	1.45	1.05	1.99	1.16	0.89	1.50

* Model 1 is adjusted for country of residence and age

** Model 2 is adjusted for country of residence, age, marital status, urban/rural area and education
