# Supplementary material for: Socioeconomic inequality in the prevalence of noncommunicable diseases in low- and middle-income countries: Results from the World Health Survey
Source: BMC Public Health. 2012 Jun 22;12:474. doi: 10.1186/1471-2458-12-474 (PMC3490890; doi:10.1186/1471-2458-12-474)
Supplement: Additional file 6 — Crude prevalence (%) of non-communicable diseases among adults aged 18 or higher living in 41 low- and middle-income countries, by wealth, World Health Survey 2002–04. Displays the crude prevalence rates (percentage) of each studied noncommunicable disease and comorbidity among adults (aged 18 or higher), according to wealth quintile. Data are grouped by sex and low- or middle-income country status, and represent 41 low- and middle-income countries that participated in the 2002–04 World Health Survey. [file 1471-2458-12-474-S6.pdf]

Additional file 6: Crude prevalence (%) of non-communicable diseases among adults aged 18 or higher living in 41 low- and middle-income countries, by wealth, World Health Survey 2002-04

			Angina			Arthritis			Asthma			Depression			Diabetes			Co-morbidity		
			Estimate	95% CI		Estimate	95% CI		Estimate	95% CI		Estimate	95% CI		Estimate	95% CI		Estimate	95% CI	
Men	Middle-income group	Wealth quintile 1	7.4	6.5	8.2	9.0	7.5	10.5	10.4	8.6	12.1	5.3	4.2	6.4	3.0	2.0	4.0	10.2	8.4	12.0
		Wealth quintile 2	7.2	6.4	8.0	8.5	7.2	9.8	8.0	6.6	9.4	3.8	2.8	4.9	2.5	1.7	3.3	8.1	6.8	9.5
		Wealth quintile 3	7.1	6.3	7.8	8.3	6.9	9.7	8.2	6.8	9.5	4.5	3.4	5.6	2.8	2.1	3.6	7.7	6.3	9.0
		Wealth quintile 4	7.4	6.6	8.2	7.7	6.5	8.8	6.9	5.8	8.1	4.1	3.2	5.0	3.0	2.3	3.6	6.9	5.7	8.0
		Wealth quintile 5	5.4	4.7	6.2	5.4	4.0	6.8	4.4	3.5	5.3	2.3	1.6	3.0	3.3	2.7	4.0	4.0	3.0	5.0
	Low-income group	Wealth quintile 1	13.0	11.4	14.6	5.2	4.0	6.4	7.7	6.6	8.7	6.3	5.3	7.4	0.8	0.5	1.1	7.6	6.3	8.9
		Wealth quintile 2	12.0	10.6	13.5	6.2	5.1	7.4	6.7	5.6	7.8	5.8	4.8	6.8	1.4	0.9	1.9	7.2	6.1	8.2
		Wealth quintile 3	10.5	9.0	12.0	4.9	3.9	5.9	5.9	4.8	7.1	5.3	4.3	6.4	1.8	1.1	2.4	5.8	4.7	6.8
		Wealth quintile 4	8.4	7.0	9.7	4.1	3.3	4.9	5.0	4.2	5.9	5.6	3.5	7.6	1.8	1.2	2.3	4.2	3.4	5.1
		Wealth quintile 5	6.1	5.1	7.1	3.7	2.8	4.5	3.7	3.1	4.3	3.5	2.8	4.2	3.4	2.7	4.2	3.3	2.6	3.9
Women	Middle-income group	Wealth quintile 1	26.9	24.3	29.5	12.0	10.3	13.6	12.0	10.6	13.4	9.4	7.9	11.0	5.7	4.6	6.8	16.1	14.3	17.9
		Wealth quintile 2	22.9	20.7	25.0	13.0	11.3	14.8	9.7	8.5	11.0	6.9	5.6	8.2	4.7	3.8	5.7	14.4	12.6	16.3
		Wealth quintile 3	17.8	16.0	19.5	10.8	9.5	12.1	9.2	7.6	10.9	6.9	5.6	8.3	3.8	3.0	4.6	11.1	9.7	12.6
		Wealth quintile 4	15.6	13.7	17.5	10.1	8.7	11.5	6.0	4.7	7.3	6.7	5.4	8.0	4.9	3.9	5.8	10.2	8.5	11.9
		Wealth quintile 5	12.6	10.5	14.6	9.4	7.9	10.9	6.7	4.6	8.8	4.0	3.2	4.7	5.0	3.2	6.8	8.1	6.1	10.1
	Low-income group	Wealth quintile 1	18.6	16.6	20.6	8.0	6.5	9.4	5.9	4.8	7.0	10.3	8.7	11.9	0.8	0.4	1.2	9.3	8.0	10.7
		Wealth quintile 2	17.4	15.8	19.0	7.7	6.6	8.8	6.6	5.6	7.6	9.2	7.9	10.5	1.6	0.9	2.2	9.3	8.1	10.6
		Wealth quintile 3	15.4	13.9	16.9	8.4	7.1	9.6	5.9	4.9	7.0	9.7	8.2	11.3	1.4	0.9	1.8	8.2	7.1	9.3
		Wealth quintile 4	13.7	11.9	15.5	6.8	5.7	7.9	5.4	4.5	6.3	8.7	7.5	9.9	2.8	1.9	3.7	7.7	6.6	8.9
		Wealth quintile 5	12.6	11.0	14.3	5.7	4.8	6.6	4.9	4.0	5.7	5.3	4.3	6.2	3.4	2.6	4.2	6.1	5.0	7.2
