# Supplementary material for: Socioeconomic inequality in the prevalence of noncommunicable diseases in low- and middle-income countries: Results from the World Health Survey
Source: BMC Public Health. 2012 Jun 22;12:474. doi: 10.1186/1471-2458-12-474 (PMC3490890; doi:10.1186/1471-2458-12-474)
Supplement: Additional file 7 — Education-related relative inequality in noncommunicable disease prevalence among adults aged 18 or higher living in 41 low- and middle-income countries, World Health Survey 2002–04. Displays the relative index of inequality and corresponding 95% confidence interval for each studied noncommunicable disease and comorbidity among adults (aged 18 or higher), according to education level. Data are grouped by sex and low- or middle-income country status, and represent 41 low- and middle-income countries that participated in the 2002–04 World Health Survey. Model 1 data are adjusted for country of residence and age; model 2 data are adjusted for country of residence, age, marital status, urban/rural area and wealth. [file 1471-2458-12-474-S7.pdf]

Additional file 7: Education-related relative inequality in noncommunicable disease prevalence among adults aged 18 or higher living in 41 low- and middle-income countries, World Health Survey 2002-04

| | | Men | | | | | | Women | | | | | |
|---|---|---|---|---|---|---|---|---|---|---|---|---|---|
| | | **Middle-income group** | | | **Low-income group** | | | **Middle-income group** | | | **Low-income group** | | |
| | | **Estimate** | **95%CI** | | **Estimate** | **95%CI** | | **Estimate** | **95%CI** | | **Estimate** | **95%CI** | |
| **Angina** | Model 1 | 1.52 | 1.19 | 1.93 | 2.20 | 1.70 | 2.86 | 1.35 | 1.10 | 1.64 | 1.66 | 1.35 | 2.06 |
| | Model 2 | 1.09 | 0.83 | 1.42 | 1.54 | 1.14 | 2.06 | 1.05 | 0.87 | 1.28 | 1.29 | 1.01 | 1.63 |
| **Arthritis** | Model 1 | 1.20 | 0.88 | 1.63 | 1.86 | 1.24 | 2.80 | 1.00 | 0.79 | 1.27 | 1.43 | 1.02 | 2.00 |
| | Model 1 | 0.97 | 0.68 | 1.39 | 1.60 | 1.03 | 2.50 | 0.94 | 0.73 | 1.20 | 1.18 | 0.79 | 1.75 |
| **Asthma** | Model 2 | 2.04 | 1.47 | 2.85 | 2.08 | 1.52 | 2.86 | 1.74 | 1.12 | 2.69 | 1.39 | 0.98 | 1.98 |
| | Model 1 | 1.41 | 0.99 | 2.02 | 1.54 | 1.02 | 2.31 | 1.41 | 0.95 | 2.08 | 1.26 | 0.87 | 1.82 |
| **Depression** | Model 1 | 1.51 | 0.96 | 2.38 | 1.45 | 0.91 | 2.29 | 1.58 | 1.07 | 2.33 | 2.19 | 1.68 | 2.87 |
| | Model 2 | 1.12 | 0.65 | 1.93 | 1.06 | 0.63 | 1.77 | 1.23 | 0.81 | 1.87 | 1.71 | 1.22 | 2.39 |
| **Diabetes** | Model 1 | 0.70 | 0.45 | 1.08 | 0.18 | 0.12 | 0.29 | 1.07 | 0.66 | 1.72 | 0.17 | 0.10 | 0.31 |
| | Model 2 | 0.98 | 0.60 | 1.61 | 0.36 | 0.20 | 0.64 | 1.44 | 0.96 | 2.17 | 0.44 | 0.22 | 0.88 |
| **Co-morbidity** | Model 1 | 1.56 | 1.10 | 2.20 | 2.46 | 1.78 | 3.41 | 1.39 | 1.04 | 1.87 | 1.97 | 1.44 | 2.71 |
| | Model 2 | 1.09 | 0.74 | 1.61 | 1.66 | 1.12 | 2.45 | 1.18 | 0.90 | 1.54 | 1.64 | 1.13 | 2.39 |

* Model 1 is adjusted for country of residence and age

** Model 2 is adjusted for country of residence, age, marital status, urban/rural area and wealth
