# Supplementary material for: Socioeconomic inequality in the prevalence of noncommunicable diseases in low- and middle-income countries: Results from the World Health Survey
Source: BMC Public Health. 2012 Jun 22;12:474. doi: 10.1186/1471-2458-12-474 (PMC3490890; doi:10.1186/1471-2458-12-474)
Supplement: Additional file 8 — Crude prevalence (%) of noncommunicable diseases among adults aged 18 or higher living in 41 low- and middle-income countries, by education, World Health Survey 2002–04. Displays the crude prevalence rates (percentage) of each studied noncommunicable disease and comorbidity among adults (aged 18 or higher), according to education level. Data are grouped by sex and low- or middle-income country status, and represent 41 low- and middle-income countries that participated in the 2002–04 World Health Survey. [file 1471-2458-12-474-S8.pdf]

Additional file 8: Crude prevalence (%) of noncommunicable diseases among adults aged 18 or higher living in 41 low- and middle-income countries, by education, World Health Survey 2002-04

			Angina			Arthritis			Asthma			Depression			Diabetes			Co-morbidity		
			Estimate	95% CI		Estimate	95% CI		Estimate	95% CI		Estimate	95% CI		Estimate	95% CI		Estimate	95% CI	
Men	Middle-income group	No formal schooling	8.2	6.8	9.7	10.9	8.7	13.1	15.1	12.1	18.0	10.1	7.4	12.8	5.8	3.7	8.0	15.5	12.5	18.5
		Less than primary school	8.6	7.3	9.8	9.3	7.7	10.9	9.5	7.7	11.2	4.2	3.1	5.3	3.4	2.5	4.3	9.3	7.7	10.9
		Primary school completed	6.4	5.5	7.4	7.7	6.6	8.9	8.2	7.2	9.2	4.9	3.9	5.9	3.9	3.0	4.9	8.2	7.1	9.4
		Secondary/high school completed	6.4	5.8	7.0	7.0	6.0	7.9	6.4	5.5	7.4	2.9	2.3	3.5	2.3	1.9	2.8	5.5	4.7	6.3
		College completed or above	5.8	4.8	6.7	7.6	6.0	9.2	5.6	4.1	7.1	3.4	2.3	4.5	2.2	1.6	2.9	6.7	5.1	8.3
	Low-income group	No formal schooling	14.3	12.9	15.7	6.9	5.9	7.8	8.3	7.2	9.3	7.2	6.2	8.1	1.3	0.9	1.7	8.5	7.4	9.5
		Less than primary school	11.3	9.8	12.9	5.3	4.3	6.3	6.6	5.4	7.8	5.8	4.7	6.8	2.2	1.4	3.0	7.2	5.9	8.5
		Primary school completed	8.1	7.1	9.2	4.0	3.2	4.9	5.1	4.2	5.9	4.8	3.9	5.7	2.0	1.3	2.6	4.4	3.6	5.2
		Secondary/high school completed	6.9	5.9	8.0	3.6	2.7	4.4	3.4	2.7	4.1	3.0	2.3	3.7	1.7	1.3	2.1	3.1	2.4	3.8
		College completed or above	5.2	3.2	7.1	2.2	1.3	3.1	4.2	2.8	5.7	6.3	1.5	11.1	3.7	2.3	5.1	2.7	1.5	3.8
Women	Middle-income group	No formal schooling	24.3	21.7	26.8	14.7	12.9	16.5	12.7	10.6	14.8	16.3	13.8	18.7	9.1	7.5	10.8	20.8	18.5	23.1
		Less than primary school	23.9	21.2	26.6	12.7	10.6	14.7	13.0	10.0	15.9	8.0	6.4	9.7	7.9	6.2	9.6	15.5	13.1	18.0
		Primary school completed	18.8	17.1	20.5	10.8	9.6	12.0	8.6	7.6	9.7	6.7	5.6	7.8	5.9	4.9	6.9	11.8	10.5	13.2
		Secondary/high school completed	17.9	16.3	19.5	10.0	8.9	11.2	7.6	6.7	8.6	5.2	4.4	5.9	3.5	3.0	4.1	10.2	8.8	11.5
		College completed or above	16.6	14.2	18.9	10.9	9.2	12.6	7.2	5.0	9.4	5.2	3.9	6.6	3.6	1.7	5.6	9.9	7.7	12.1
	Low-income group	No formal schooling	19.6	18.2	20.9	9.4	8.5	10.2	7.1	6.4	7.9	11.4	10.4	12.5	2.2	1.7	2.7	11.1	10.2	12.0
		Less than primary school	14.7	13.0	16.4	7.0	5.9	8.2	5.3	4.4	6.3	8.1	6.7	9.5	1.8	1.0	2.5	7.6	6.3	8.8
		Primary school completed	11.5	10.2	12.8	5.2	4.3	6.1	4.6	3.6	5.5	6.5	5.6	7.5	2.2	1.4	3.0	5.1	4.3	6.0
		Secondary/high school completed	9.8	8.3	11.3	4.2	3.1	5.3	3.5	2.7	4.4	3.9	3.0	4.8	1.5	0.9	2.1	3.7	2.8	4.6
		College completed or above	6.0	3.8	8.1	3.8	1.9	5.6	2.8	1.3	4.2	2.4	1.1	3.8	1.6	0.7	2.5	2.1	1.1	3.2
